# A Comparison of Guided Bone Regeneration vs. the Shell Technique Using Xenogeneic Bone Blocks in Horizontal Bone Defects: A Randomized Clinical Trial

**DOI:** 10.3390/dj12050137

**Published:** 2024-05-09

**Authors:** Paolo De Angelis, Camilla Cavalcanti, Paolo Francesco Manicone, Margherita Giorgia Liguori, Edoardo Rella, Giuseppe De Rosa, Alberto Palmieri, Antonio D’Addona

**Affiliations:** 1 Division of Oral Surgery and Implantology, Institute of Clinical Dentistry, Oral Surgery, and Implantology Unit, Department of Head and Neck, A. Gemelli University Hospital Foundation (IRCCS) Catholic University of the Sacred Heart, 00168 Rome, Italy; dr.paolodeangelis@gmail.com (P.D.A.); cami97cavalcanti@gmail.com (C.C.); paolofrancesco.manicone@unicatt.it (P.F.M.); giuseppederosa1410@gmail.com (G.D.R.); alberto.pamieri81@gmail.com (A.P.); antonio.daddona@unicatt.it (A.D.); 2 Department of Life, Health and Environmental Sciences, University of L’Aquila, 67100 L’Aquila, Italy; margheritagliguori@gmail.com

**Keywords:** bone graft, guided bone regeneration, biomaterials, randomized clinical trial, bone substitutes, shell technique

## Abstract

In cases of severe horizontal atrophy, implant placement requires bone reconstruction procedures. The aim of this randomized controlled trial is to compare the outcomes of bone augmentation with simultaneous implant placement using the shell technique to the outcomes of guided bone regeneration (GBR) in cases of severely horizontal bone atrophy. This study was designed as a monocentric, parallel-group, randomized controlled trial with a six-month follow-up. Among the primary outcomes of this study, peri-implant bone regeneration and peri-implant bone defect closure were selected. Forty-four patients were recruited and equally divided between two groups. In the GRB group, a horizontal regeneration of 2.31 ± 0.23 mm was observed opposed to a horizontal regeneration of 2.36 ± 0.17 mm in the shell group (*p* = 0.87). A volumetric increase was observed in both groups, with an increase of 0.30 ± 0.12 cm^3^ in the GBR group and an increase of 0.39 ± 0.09 cm^3^ in the shell group, highlighting a significant difference between the two groups (*p* = 0.02)**.** In conclusion, bone augmentation with simultaneous implant placement using the shell technique or guided bone regeneration in horizontal bone atrophy are both predictable therapeutic options.

## 1. Introduction

The management of edentulous patients with implant-prosthetic rehabilitations is now considered a reliable procedure with predictable long-term results [1]. Nevertheless, in cases of severe bone deficiency, augmentation procedures may become necessary to ensure proper prosthetically guided three-dimensional implant placement and to avoid the risk of jeopardizing aesthetic and functional outcomes [2]. The augmentation of hard tissue volume can be achieved through various surgical techniques based on the use of particulate and block grafting materials, which may be associated with resorbable and non-resorbable membranes [3,4]. Although GBR by means of bone grafts and resorbable membranes is a commonly adopted and documented technique, in relation to conditions in which it is not possible to obtain the volume stability of grafting materials, the scientific literature indicates the use of membrane-supporting materials as mandatory [5]. Also, different techniques for bone reconstructions by means of block grafts have been described. The “three-dimensional” reconstruction technique or shell technique, introduced by Khoury in 2004, involves the placement of thin cortical bone blocks in order to restore the defect walls, filling the resulting gaps with autogenous bone chips [6]. This method allows for accelerated vascularization in the inner part of the reconstruction, and by providing stability to the bone plate, it reduces bone resorption, thus overcoming the limits of previous techniques such as the onlay and inlay techniques [7]. Actually, as evidenced by numerous studies, membrane-surrounded autogenous block grafts provide the best degree of dimensional stability, particularly if protected by collagen membranes. Compared to previous membranes, this membrane is more manageable, allows for less extended flap designs, and is associated with lower rates of wound dehiscence [8,9]. In conjunction with membranes, bone blocks provide a primary advantage, which is the ability to avoid membrane collapse, leading to reduced reabsorption [10]. Furthermore, thanks to this reduced resorption rate, simultaneous implant placement is also possible in the case of vertical bone augmentation [11].

Autogenous block grafts are considered the gold standard by many clinicians given their osteoinduction, osteoconduction, and osteogenic properties [12]. Unfortunately, disadvantages have also been described: an increased morbidity due to the presence of a donor site (usually the retromolar region or the symphysis), limited graft availability, a significant resorption rate (18–60%) compared with the initial volume, and a risk of postoperative complications [13,14]. With the aim of avoiding intraoral bone harvesting, xenogeneic bone blocks have been introduced into clinical practice as an alternative to autogenous bone blocks, and they have been evaluated in both animal and human studies [14,15,16]. It has been described that they offer adequate mechanical stability, and they are able to counterbalance the collapse of the membrane and of the overlying soft tissue [16]. Currently, the scientific literature has not yet expressed a unanimous opinion on the use of xenogeneic bone grafts, even if it seems that they allow a similar bone gain and cause less resorption than autogenous bone blocks [17].

Furthermore, according to a recent systematic review, postoperative dimensional changes in the augmented bone seem to diminish with the use of barrier membrane coverage [18].

The aim of this RCT is to compare the outcomes of bone augmentation with simultaneous implant placement using a combination of xenogeneic bone blocks and a resorbable membrane to the results of guided bone regeneration in severely horizontal bone atrophy in terms of peri-implant defect closure and bone regeneration.

## 2. Materials and Methods

This study was designed as a monocentric, parallel-group, randomized controlled trial with a six-month follow-up; it was conducted at the Catholic University of the Sacred Heart between May 2022 and July 2022. The study protocol was approved by the Institutional Review Board of the Catholic University of the Sacred Heart (0016547/22). This study was conducted following the guidelines of the Helsinki Declaration of 1975, as revised in 2000.

All the patients who participated in the study were informed about the aims and methods of the research and signed informed consent forms. CONSORT guidelines were followed.

All patients involved in the study were informed about its aims and methods and signed informed consent forms. This study was registered at Clinicaltrials.gov (0016547/22) (accesssed on 13 December 2023).

### 2.1. Participants

Patients in need of an implant restoration were enrolled at the Department of Oral Surgery and Implantology at the Catholic University of the Sacred Heart. Two different treatment modalities to treat horizontal peri-implant bone defects were compared: GBR with a resorbable membrane and the shell technique with a xenogeneic bone block. Patients were selected on the basis of the following inclusion criteria:

1. Patients in need of implant-supported rehabilitation;
2. Horizontal bone atrophy allowing for implant placement with a torque of at least 20 Ncm;
3. Bone augmentation procedure simultaneous to implant placement;
4. Healthy patients (absence of contraindications for implant surgery and bone augmentation procedures);
5. Full-mouth plaque score (FMPS) and full-mouth bleeding score (FMBS) ≤ 15%;
6. Sufficient mesiodistal and interocclusal space.

The exclusion criteria were the following:

1. An American Society of Anesthesiologists physical status classification of ≥III;
2. Systemic diseases;
3. Untreated periodontal disease;
4. Smoking;
5. Uncontrolled diabetes;
6. Excessive alcohol consumption;
7. No residual keratinized tissue at the experimental area.

### 2.2. Outcomes

Among the primary outcomes of this study, peri-implant bone regeneration and peri-implant bone defect closure were selected. The outcomes listed as secondary were the bone volume gain, the stability of periodontal parameters, the width of the keratinized mucosa, wound healing, pain, and the incidence of post-surgical complications.

### 2.3. Interventions

**Figure 1:**
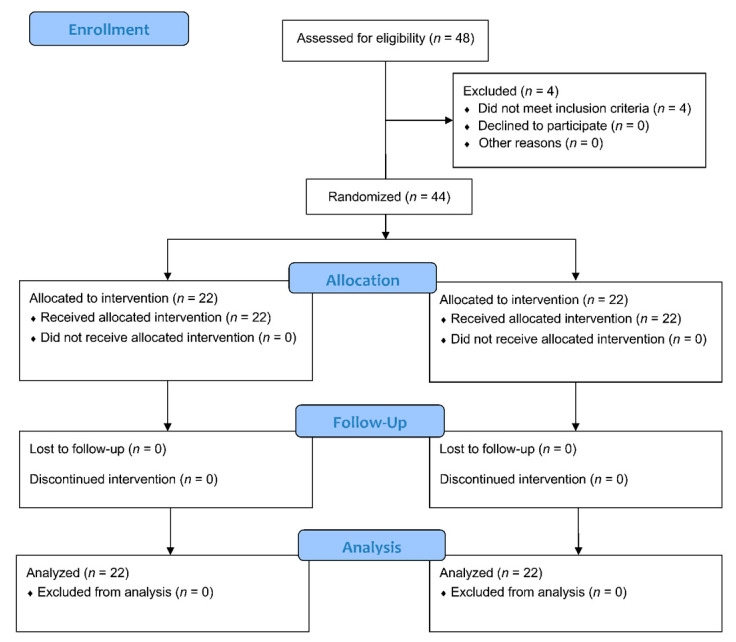
The workflow followed in the present study.

Forty-four patients who fulfilled the above criteria and offered consent were enrolled in this study. Patients were randomly assigned to one of two groups, the GBR group (*n* = 22) or shell technique group (*n* = 22), using the block randomization method with six samples in each block. The workflow of the present study is explained in Figure 1.

A trained clinician performed all of the surgeries using standardized procedures and recorded the intraoperative measures. All of the clinical and radiographic outcomes of the treatment were assessed by a calibrated examiner who was blinded with respect to the surgical procedures.

**Figure 2:**
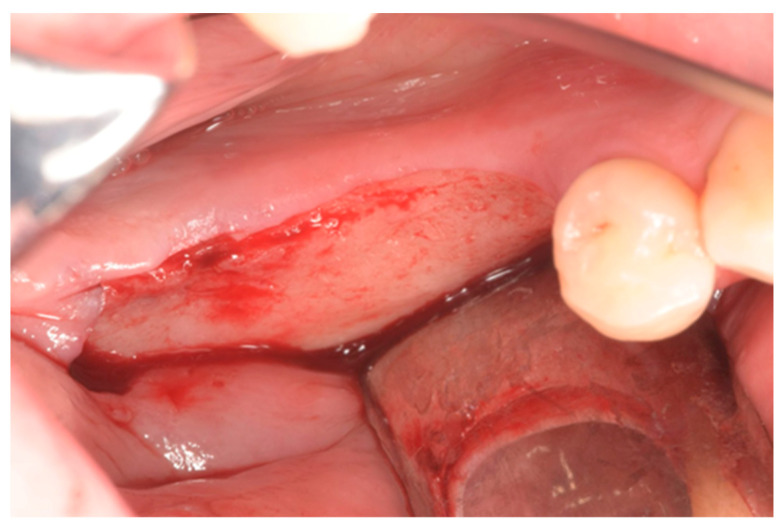
Occlusal view of the site.

Prior to the surgery, patients were given antibiotic treatment (2 doses of 1 g of amoxicillin clavulanate). The skin around the mouth was disinfected using sterile gauze attached to Klemmer forceps and soaked in povidone-iodine solution. Patients were then draped with TNT sheets, and mucous membranes were cleaned using gauze soaked in 0.2% chlorhexidine solution. The surgery was conducted under local anesthesia (using articaine 4% with epinephrine at a ratio of 1:100,000). A horizontal incision was made along the gingiva in the lower jaw and slightly towards the vestibular in the upper jaw, starting from the back of one tooth to the front of the adjacent tooth (if the adjacent tooth was absent, a safe distance was kept from the atrophic area). The incision was intrasulcular and followed the margin along the surface of the teeth. Additional releasing incisions were made if necessary. A full thickness flap was lifted, exposing the bone, which was then carefully cleaned using a curette (Figure 2).

**Figure 3:**
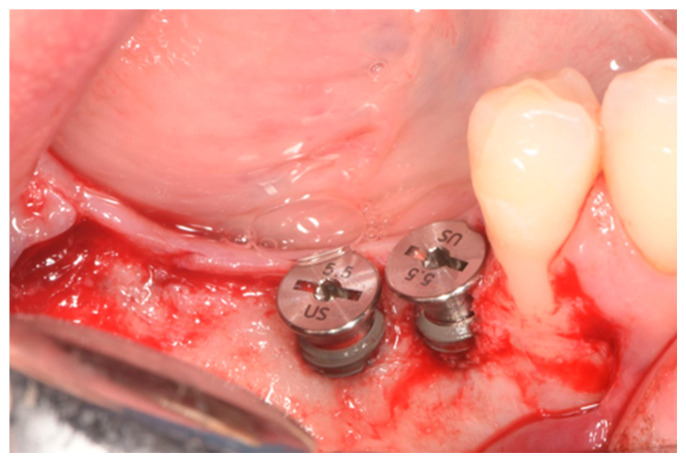
Lateral view after implant placement with bone dehiscence.

The nearby teeth were meticulously cleaned using both ultrasonic and manual tools. Following the manufacturer’s instructions, bone-level implants ranging from 8 to 12 mm in length and 3.3 to 4.8 mm in diameter were carefully inserted (see Figure 3). Primary stability during implant placement was evaluated through torque measurement and manual examination. To promote bleeding and facilitate access to the marrow cavity, the cortical plate was perforated using a round bur.

In the GBR (guided bone regeneration) group, a resorbable cross-linked pericardium membrane (Shelter, Ubgen, Vigonza, PD, Italy) was shaped to fit the specific recipient site and secured on the lingual/palatal side using two or three fixation pins. Autogenous bone chips were harvested from areas surrounding the peri-implant defect or from a secondary surgical site (such as the retromolar area or the external oblique line of the mandible) using a bone scraper. These bone chips were then placed near the implant surface and mixed with particulate bovine bone (Rebone, Ubgen, Vigonza, PD, Italy) in a 50:50 ratio to completely fill the defect area. The pericardium membrane was then closed over the graft and secured on the buccal side using two or three titanium pins.

**Figure 4:**
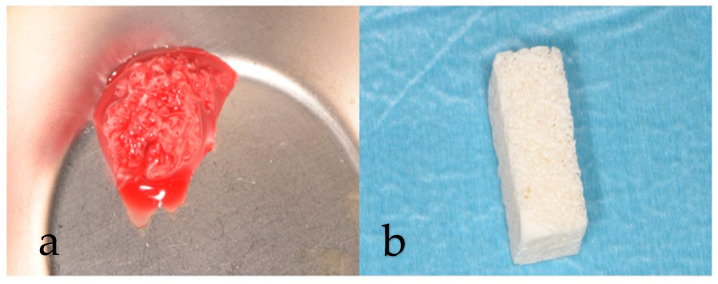
(**a**) Autogenous bone chips (**b**) Xenogeneic block graft.

**Figure 5:**
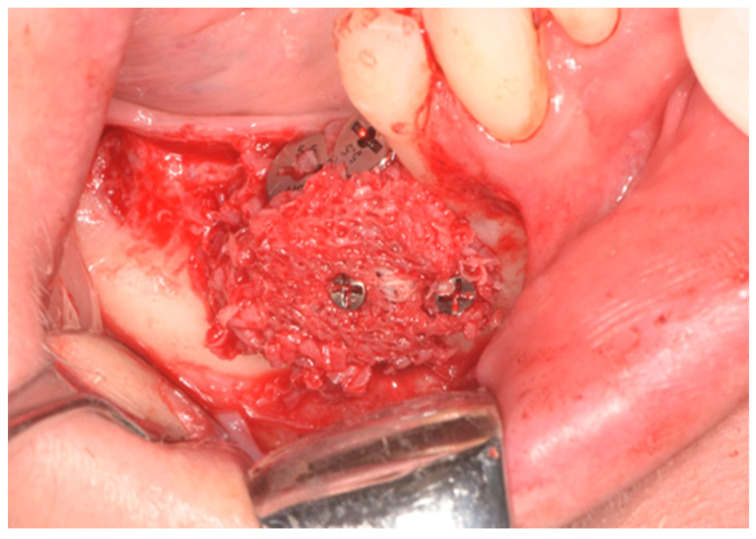
The placement of the xenograft with the shield technique with autogenous bone chips inside.

In the shell technique group, a bovine bone block (Rebone, Ubgen, Vigonza, PD, Italia) was shaped according to the recipient site and sectioned with a thickness of approximately 1.5–2 mm. This shell of xenograft was trimmed and adjusted with a round bur to remove the angles and guarantee a good fit; then, it was positioned and fixed in the host bone using osteosynthesis screws to stabilize it. The space between the shell and the alveolar bone was filled with particulate autogenous bone chips. The grafted area was covered with a cross-linked pericardium membrane (Shelter, Ubgen, Vigonza, PD, Italia) (Figure 4 and Figure 5).

Releasing incisions in the periosteum were made to ensure the mucoperiosteal flaps could be adapted without tension. The crestal incision was closed with internal horizontal mattress sutures, and single stitches were then placed on the vertical incisions and between the mattress sutures. Patients were advised to rinse their mouths twice daily with 0.2% chlorhexidine mouthwash and to complete the antibiotic course for six days. Additionally, analgesics (80 mg ketoprofen) were prescribed for the following three days based on individual requirements.

**Figure 6:**
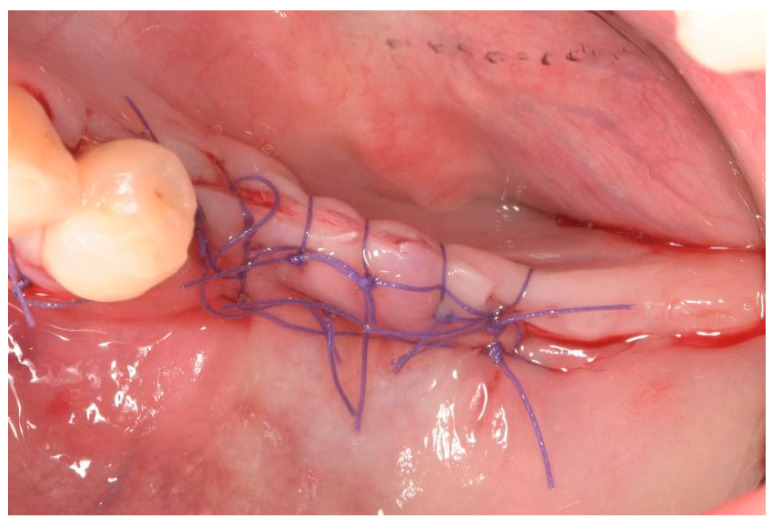
Suture of surgical site.

Patients were also instructed to avoid mechanical plaque removal in the area for a period of two weeks. Sutures were removed twenty-one days after the surgery, and all patients were included in a maintenance care program. The second surgery was performed six months later (see Figure 6).

**Figure 7:**
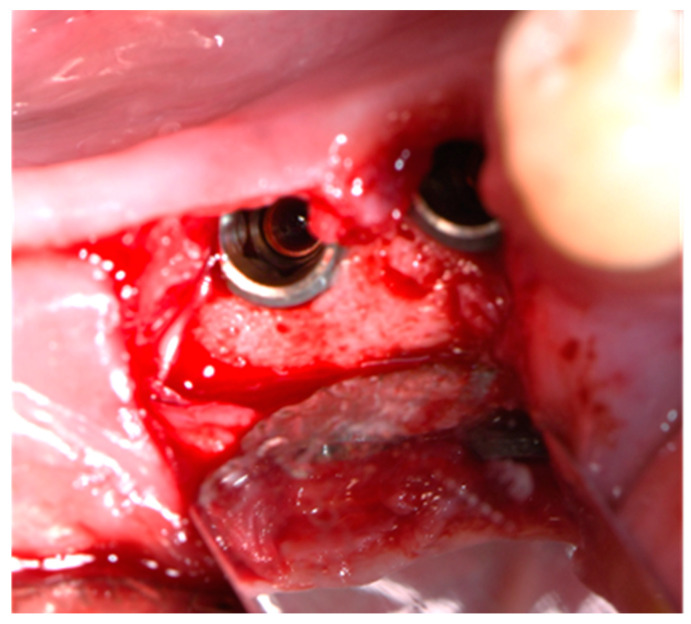
Surgical re-entry after augmentation procedure.

A cone beam CT scan was taken before the surgery to plan the treatment and after six months of healing to validate the results obtained with the surgery (Figure 7).

### 2.4. Data Collection

Patients’ information, including gender, age, and site of the graft, was recorded.

The following measurements were collected at baseline and six months after surgery at the tooth sites adjacent to the treatment area:

- The width of keratinized tissue (KT);
- Plaque index (PI);
- Gingival index (GI);
- Probing depth (PD);
- Bleeding on probing (BOP).

During the surgery, the peri-implant bone defect was measured using a periodontal probe (UNC-15), and the following parameters were documented on the clinical chart following the method outlined by Jung et al. [19]:

(1) Vertical defect height (mm), measured from the implant shoulder to the first bone-to-implant contact (BIC);
(2) Defect width (mm), measured from the mesial to the distal bone crests at the level of the implant shoulder;
(3) Horizontal defect depth (mm), measured from the bone crest to the implant surface in a perpendicular direction to the long axis of the implant at the level of the implant shoulder [20].

Wound healing progress was evaluated using the early wound healing score (EHS), which consists of three components: clinical indicators of re-epithelialization, signs of hemostasis, and evidence of inflammation. The sum of points from these three parameters determines the EHS. A perfect wound healing outcome is indicated by a score of 10 points, while the lowest possible score is 0. EHS assessments were conducted every seven days during the initial three-week period.

Peri-implant bone regeneration was evaluated at the time of surgical re-entry by measuring the amount of bone gained in millimeters using the above-described method. The occurrence of adverse events (e.g., wound infection, exposure of the graft, and soft tissue dehiscence) was recorded during the whole duration of the follow-up.

**Figure 8:**
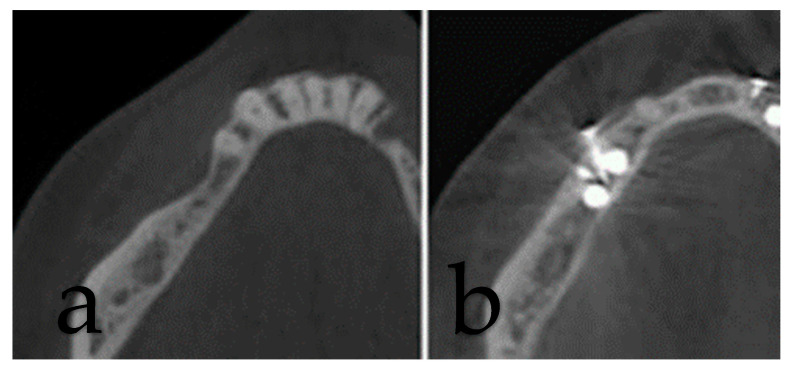
(**a**) Pre-operative evaluation of the site. (**b**) Post-operative evaluations of the site.

A volumetric analysis was performed using a software (Materialise Mimics 20.0) to evaluate hard tissue tridimensional changes in the region of interest. The bone graft was modeled using a multiple slice edit segmentation tool. The screws and implants were then segmented individually using a threshold tool (metallic high density); a Boolean subtraction tool was then used to subtract the implant and screw volume from the bone graft. Finally, a measurement tool was used to obtain the volume in mm^3^, which was then converted to cm^3^ (Figure 8).

Patient-reported outcomes were evaluated using an in-house visual analogue scale questionnaire (VAS), completed by the patients, which was administered after seven days in order to assess pain (VAS; 0 = no pain, 10 = extremely painful).

In the second surgery, which was conducted to uncover the submerged implant, tissue samples were procured for histological examination using a calibrated trephine bur. These tissue samples were then stained with hematoxylin and eosin for analysis.

### 2.5. Statistical Analysis

To keep the absolute horizontal gain as the main outcome and according to what is available in the literature, in order to reach a statistical power of 0.8 while maintaining a level of statistical significance of 0.05, a sample size of thirty-eight subjects equally divided into two groups is needed [21].

Qualitative variables were described as absolute and relative frequencies, while quantitative variables were summarized as mean and standard deviation. After assessing the data for normality using the Shapiro–Wilk test, an independent samples *t*-test was applied for parametric numerical variables to determine the significance of the difference in the reported outcomes between the GBR group and shell group. Categorical variables were analyzed with Pearson’s chi-squared test or Fisher’s exact test according to the variable distribution. All of the analyses were performed with Stata Statistical Software version 17 (StataCorp LLC, College Station, TX, USA), and a *p*-value of <0.05 was set as the threshold for statistical significance.

## 3. Results

Forty-four patients were recruited and equally divided between the two groups, with an overall mean age of 43.34 ± 8.13.

The pre-surgical variables are reported in Table 1; the post-surgical variables are reported in Table 2.

**Figure 9:**
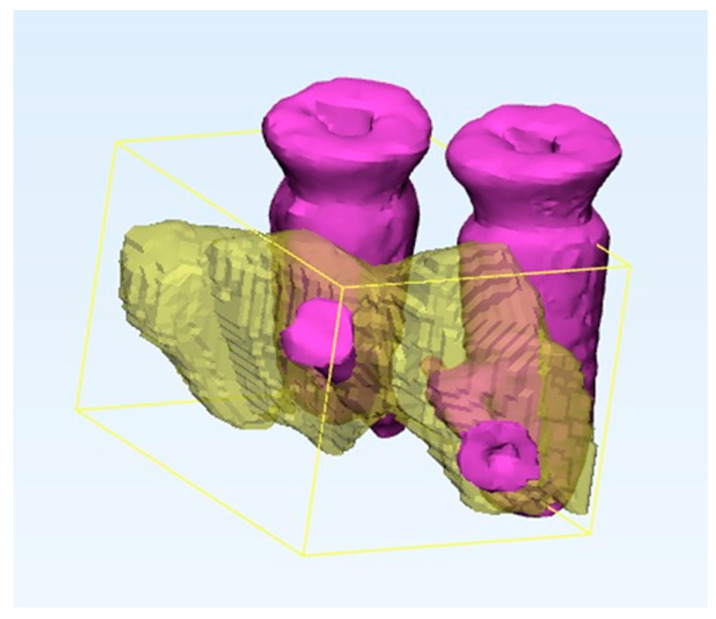
Volumetric analysis of augmented site.

In the GBR group, a vertical bone gain of 2.10 ± 0.87 mm was observed, while in the shell group, this increase amounted to 2.18 ± 0.79 mm (*p* = 0.71). The first group showed a horizontal regeneration of 2.31 ± 0.23 mm opposed to the horizontal regeneration of 2.36 ± 0.17 mm observed in the shell group (*p* = 0.87). Similarly, bone regeneration for the parameter defect width was 2.81 ± 0.21 mm in the GBR group and 3.14 ± 0.25 mm in the shell group (*p* = 0.33). None of these three differences between the two groups were statistically significant (*p* > 0.05) (Figure 8). A volumetric increase was observed in both groups, corresponding to 0.30 ± 0.12 cm^3^ in the GBR group and 0.39 ± 0.09 cm^3^ in the shell group, showing a significant difference between the two groups (*p* = 0.02) (Figure 9).

A complete defect closure was observed in nineteen cases in the GBR group (86.36%) and in twenty cases in the shell group (90.91%).

**Figure 10:**
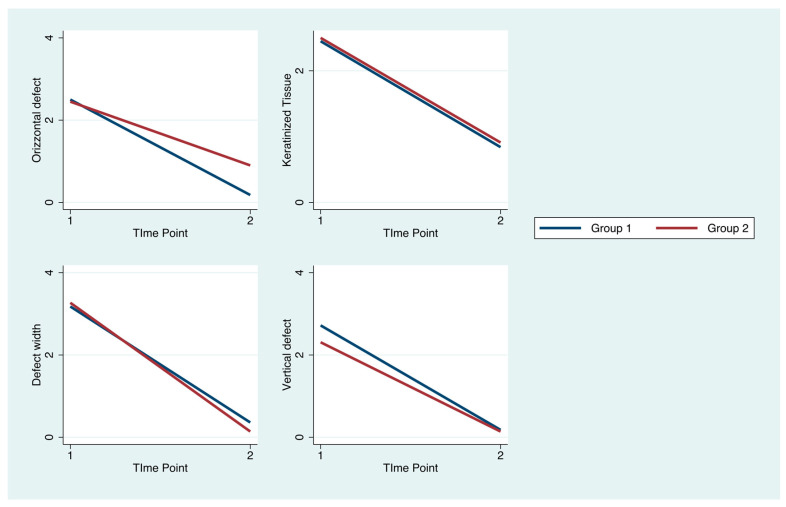
The four major variables shown at T0 and T1 in both groups: horizontal defect, keratinized tissue, defect width, and vertical defect.

A notable reduction in the width of keratinized tissues was also observed in both groups (GBR group = 1.6 ± 0.21, shell group = 1.58 ± 0.28) without significant differences between the two (*p* > 0.05). No difference between the groups was observed for any of the remaining periodontal variables (*p* > 0.05) (Figure 10).

No cases of intraoperative complications were recorded. Five postoperative complications were observed between the two groups (GBR group = 2; shell group = 3). Wound dehiscence was recorded in two cases in the GBR group and one case in the shell group. An incomplete integration of the xenogeneic block, which required a partial removal, was recorded in two cases of the shell group. Patients that had post-surgical complications were statistically more at risk of not achieving complete defect closure (*p* < 0.001).

No statistically significant differences in postoperative wound healing were observed for the first three weeks between the groups when analyzing the EHS (*p* > 0.05).

The recorded surgical pain, obtained using the VAS scale, amounted to 6.77 ± 0.20 in the GBR group and 6.68 ± 0.18 in the shell group (*p* > 0.05).

No further correlations between complete defect closure and the number of implants or keratinized tissues were investigated because of the small amount of incomplete closures, making the statistical models unreliable.

**Figure 11:**
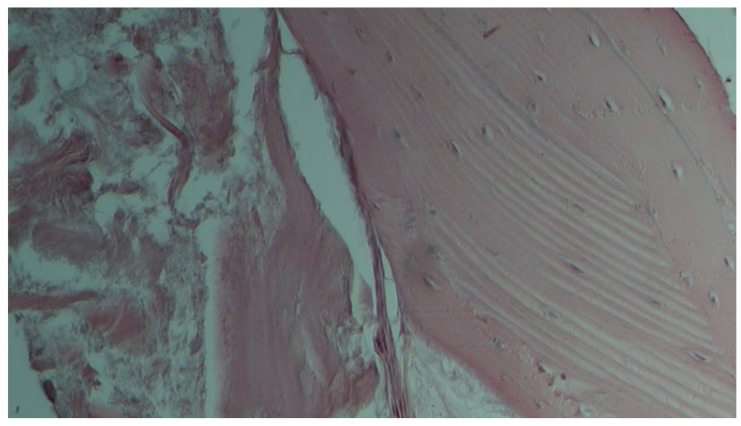
Lamellar bone tissue with havers canals and lacunae containing osteocytes. It is also possible to observe osteoblast nuclei with the synthesis of the bone matrix.

The histological analysis revealed the integration of the xenogeneic bone blocks with the recipient site in the harvested samples, with no signs of any pathological reactions (Figure 11). However, remaining xenogeneic bone substitute was observed in the samples surrounded by variable amounts of new bone and connective tissue.

## 4. Discussion

Atrophic jaws represent a challenge for clinicians when it comes to implant placement and subsequent restoration in order to fulfill patients’ requests. In cases when neither less invasive alternatives, such as narrow or short implants, nor removable prostheses were possible or accepted by a patient, several augmentation techniques have been widely documented: among these, many authors have indicated the use of autogenous bone blocks as the gold standard since they have good osteogenetic, osteoinductive, and osteoconductive properties. Nevertheless, the harvesting procedure may imply postoperative complications as well as limited availability and a higher degree of resorption compared to other grafts [18,20,21].

The 3D reconstruction technique, or shell technique, was first described by Khoury et al.; it consists of using thin cortical bone plates made by splitting cortical bone blocks harvested from the retromolar area and placing them to create a 3D place over the bone crest, which is then filled with autogenous bone chips [6,22,23].

Currently, GBR by means of both resorbable and non-resorbable membranes and a mixture of particulate autogenous and xenogeneic bone grafts has also been well documented and is considered a very predictable technique. Nevertheless, particulate bone substitutes also proved to have less stability when used in severe bone deficiencies [24].

In order to overcome the limits of membranes and reduce the discomfort associated with the harvesting of block grafts, xenogeneic bone blocks have been proposed.

The use of allogeneic blocks as shells in bone regeneration was first compared to autogenous bone blocks by Tunkel et al. in a split-mouth case report. Their aim was to reduce patients’ morbidity associated with the harvesting procedure; they combined the allogeneic bone plate with autogenous bone chips, which were collected from the contralateral augmentation, and reported no significant differences in terms of horizontal and vertical bone gain as well as resorption rates between the two groups. They observed that the combination of the principles of the shell technique and the use of an allogeneic bone block proved to be effective since it reduced the resorption rates and the postoperative morbidity that is often associated with autogenous bone transplantation [25].

However, there is still a lack of information on the use of allogeneic cortical plates for bone augmentation techniques. For this reason, the purpose of the present study is to evaluate the difference between the GBR technique and the use of cortical xenogeneic bone blocks made of bovine bone and covered by cross-linked bovine pericardium membrane in terms of both horizontal and vertical gain at 6 months as primary outcomes.

It was not possible to demonstrate significant differences between the two groups in relation to vertical and horizontal bone gain and bone regeneration of the defect width. This is in line with previous studies. Benic et al. compared the use of particulate deproteinized demineralized bovine bone mineral (DBBM) + collagen membrane (CM), block DBBM + CM, equine bone substitute block + CM, and a control group without any treatment in peri-implant defects, and they showed no significant differences between all of the GBR groups in the augmented area. Moreover, in a computed tomography investigation, the ridge dimensions were higher than those of the empty controls [16,17].

Schwartz et al. conducted a monocenter prospective clinical study to assess the clinical performance of a collagenated xenogeneic bone block (CXBB) for lateral ridge augmentation; at 24 weeks, at the time of implant insertion, the mean ridge width was 3.88 ± 1.75 mm with homogeneous osseous organization, as proven by a histological analysis. The same clinical study from the same group investigated the implant survival rate at 4.5 years, obtaining a 100% success after 2.5 years but a decrease at the last follow-up caused by patient drop-outs [26,27].

Also, the clinical study by Ortiz-Vigòn et al. [28] evaluated the safety of CXBB for lateral alveolar crest augmentation, thus obtaining an average ridge width increase of 4.12 mm at re-entry. Nevertheless, 5 out of 14 patients experienced soft tissue dehiscence and a high incidence of early implant loss (30%).

However, even if the results of our study, for what concerns the dimensional change, is in line with the literature about xenogeneic bone blocks, it should be underlined that the previously mentioned studies used xenogeneic bone blocks to perform an onlay bone grafting procedure and not to perform the shell technique.

The incidence of soft tissue dehiscences, which might have affected the results of the following implant therapy, has been positively correlated with the number of blocks used. In fact, in the narrowest crests, more than one bone block was used, thus complicating the primary wound closure of the flap over the augmentation area [28].

In the present clinical study, no cases of intraoperative complications were recorded, and five total postoperative complications were observed in the two groups, as previously reported.

However, no statistically significant differences in postoperative wound healing (EHS) were observed in either of the two groups for the first three weeks.

Also, in order to reduce the high resorption rate of autogenous block grafts, various barrier membranes were eventually applied. A recent systematic review by Gorgis et al. concluded that the gain in lateral alveolar ridge augmentation was not statistically affected by the use of a barrier membrane; however, it was associated with lower resorption rates than the use of a block graft alone [19].

In the present study, a cross-linked pericardium bovine membrane was used. The degree of cross-linking collagen membranes affects the resorption rates of both the graft and the membrane itself, thus obtaining a lower degradation and a longer stability of the scaffold [29,30].

The main advantage of xenogeneic bone blocks can be said to be their mechanical stability. Nevertheless, it might be of greater importance to investigate the quality of the regenerated bone, rather than the quantity. In their pilot investigation on xenogeneic block grafts, Benic et al. reported that histological and histomorphometric analyses showed a small amount of new bone, especially in lateral sections, while in the majority of the central ones, only a minor portion of the previously exposed implant surface was in contact with the surrounding bone. Moreover, bovine granulate reached higher values of bone-to-implant contact (BIC) compared to xenogeneic bone blocks; the reason for this result may be explained by the ingrowth of blood vessels that could take place more in the porous macrostructure of the granules rather than on the onlay block graft area, where the growth of new bone was limited to peripheral areas in contact with the cancellous bone [15,25].

In order to overcome the limit of the reduced vascularization and the limited new bone formation of xenogeneic onlay block grafts, the use of these blocks as shells has been proposed to create a space, filled with autogenous bone particles, where a blood clot can be stabilized, thus enhancing the quality of the regenerated bone around the implants [11].

The results from the histological analysis of the samples were optimal in relation to both techniques. The results of the histological analysis of the samples related to both techniques showed a good integration of the xenogeneic bone blocks, with their remnants being surrounded by new bone and connective tissue. However, it should be outlined that cases of incomplete integration of the xenogeneic block in the peripheral part of the regenerated area were recorded, requiring a partial removal. These cases were statistically more at risk of not achieving complete defect closure.

As previously mentioned, the main advantages of xenogeneic bone blocks are reduced morbidity, the limited amount available, and, last but not least, the reduced surgery time. Tunkel et al. [11], in a recent prospective–observational clinical trial, compared allogeneic and autogenous shell techniques in bone augmentation; their main finding was that the length of surgery was significantly longer if the surgeon had to harvest autogenous bone. Also, the intra and postoperative complications were similar in both groups. Nevertheless, a few complications were reported, like fracture or exposure of the graft, flap dehiscence, and loose osteosynthesis screws. Hence, it is recommended to perform bone augmentation techniques by means of good skill and knowledge because the use of xenogeneic bone blocks should not be considered as a simplification of the surgical technique [25,31].

The limitations of the present study are represented by the heterogeneity, the size of the sample, and the short follow-up time. This led to the absence of long-term peri-implant outcomes to monitor the stability of the regenerated bone. For these reasons, the results should be interpreted with caution, and other studies with homogenous samples, longer follow-ups, and histological evaluations are required.

## 5. Conclusions

Bone augmentation with simultaneous implant placement using the shell technique and guided bone regeneration in horizontal bone atrophy are both predictable therapeutic options. A similar bone gain and defect closure without significant clinical differences were observed between the two groups, even if a greater volume increase was recorded in the shell group. These results should be interpreted with caution, and other randomized clinical trials with homogenous samples and longer follow-ups are desirable.

## Figures and Tables

**Table 1:** Characteristics of defects at baseline. (GBR = guided bone regeneration.)

	GBR Group	Shell Group
Vertical dimension of defect (mm)	2.27 ± 0.82	2.31 ± 0.83
Horizontal dimension of defect (mm)	2.5 ± 0.85	2.45 ± 0.67
Defect width (mm)	3.18 ± 0.9	3.27 ± 1.08
Width of keratinized tissue (mm)	2.45 ± 1.18	2.5 ± 1.44

**Table 2:** Characteristics of defects at T1. (GBR = guided bone regeneration.)

	GBR Group	Shell Group
Vertical dimension of defect (mm)	0.17 ± 0.53	0.13 ± 0.46
Horizontal dimension of defect (mm)	0.19 ± 0.48	0.09 ± 0.29
Defect width (mm)	0.36 ± 1	0.13 ± 0.46
Width of keratinized tissue (mm)	0.86 ± 0.64	0.91 ± 0.52

## Data Availability

Data are available on request.
